# Study on Chaiyuwendan decoction’s inhibition of hippocampal neuron apoptosis to alleviating depression by activating the AKT/CREB pathway

**DOI:** 10.3389/fphar.2025.1610899

**Published:** 2025-10-27

**Authors:** Shaoliang Zhang, Sijie Cheng, Huiqing Liang, Yan Qiu, Jie Ren, Yv Huang, Bangyan Zhou, Chunrong Li, Jingmin Zhang, Tianxiang Wang, Liuyi Wang, Ruobing Liu, Qi Liu, Hongli Zhuang, Penghua Lai, Shaodong Chen

**Affiliations:** ^1^ School of Medicine, Xiamen University, Xiamen, Fujian, China; ^2^ Liver Disease Center, Xiamen Hospital of Traditional Chinese Medicine, Xiamen, Fujian, China; ^3^ School of Traditional Chinese Medicine, Beijing University of Chinese Medicine, Beijing, China; ^4^ Department of Traditional Chinese Medicine, First Affiliated Hospital of Xiamen University, Xiamen, Fujian, China; ^5^ School of Traditional Chinese Medicine, Xiamen University Malaysia, Sepang, Malaysia

**Keywords:** Chaiyuwendan decoction, depression, hippocampal neuron, apoptosis, AKT/CREB signaling pathway

## Abstract

**Background:**

Hippocampal neuron damage is closely related to depression, and apoptosis is an important pathway for neuronal damage. Depression belongs to Yubing (depressive disease) in traditional Chinese medicine theory. Chaiyuwendan decoction (CYWD) has significant clinical efficacy in treating depression, however, its specific mechanism is still unclear. This study aims to explore the potential pharmacological active substances and key therapeutic targets of CYWD in treating depression from signaling pathways related with apoptosis.

**Methods:**

HPLC-MS method was used to detect the key components of CYWD. The mouse depression model was established by CORT injection. Depressive mice were administered CYWD at low, medium and high doses. Behavioral experiment, neurotransmitters in hippocampus and serum, hippocampal HE staining and Nissl staining were tested in order to evaluate the effects of CYWD in antidepressant and anti nerve damage. The potential targets and signaling pathways for CYWD against depression were predicted through network pharmacology and molecular docking. CORT intervention was used to establish a neuronal apoptosis model of HT22. The effect of CYWD on HT22 were evaluated using CCK-8 proliferation, Nissl staining and apoptotic assays by flow cytometry. According to the predicted results, Western blot and Immunofluorescence assay were used to detect AKT, pAKT, CREB, pCREB and apoptosis-related proteins in hippocampus and HT22 cells.

**Results:**

134 active components in CYWD were identified, including chlorogenic acid, coumaric acid, rutinoside, naringin, and hesperidin. The *in vivo* studies showed that CYWD treatment improved mice’ depression-associated behaviors, decreased 5-HT, DA and NE while increased ACTH, reduced hippocampal neuronal damage. Furthermore, the AKT-CREB pathway to inhibit neuronal apoptosis was predicted as the potential key target for CYWD treatment of depression by network pharmacology methods and molecular docking. Subsequently, *in vitro* and *in vivo* experiments confirmed that CYWD can inhibit the pro-apoptotic proteins Bax and Caspase 3 by increasing AKT and CREB, and increase anti-apoptotic protein Bcl-2, thereby inhibiting CORT induced apoptosis of mouse hippocampal neurons.

**Conclusion:**

CYWD can alleviate depression through protecting hippocampal neurons by activating the AKT-CREB signaling pathway, increasing the proportion of anti apoptotic proteins. These findings provided a valuable reference to CYWD as a promising alternative against depression.

## 1 Introduction

Depression is a mental health disorder characterized by significant and persistent low mood, slow reactions, decreased memory, and even suicidal tendencies, with high rates of disability and mortality (Depressive disorder (depression), 2023; Palay et al., 2019). About 3.8% of the world’s population is affected by depression, and depression is expected to be the first disease on the global economic burden by 2030 (Depressive disorder (depression), 2023). Chemical antidepressants are the main treatment for depression, but studies have shown that existing antidepressants have poor effects, with only one-third of patients showing partial efficacy and accompanied by adverse reactions (Depressive disorder (depression), 2023).Therefore, it is urgent to seek safe and effective antidepressants.

Chaiyuwendan decoction (CYWD) is composed of a famous traditional Chinese medicine formula called Wendan Decoction and *couplet medicines* of *Bupleurum chinense DC.* (Chaih Hu) and *Curcuma wenyujin* Y.H.Chen et C.Ling (Yujin) which are used together to soothe the liver and relieve depression. CYWD has achieved good therapeutic effects in treating depression (Li, 2008). Although some experiments have confirmed the antidepressant effect of CYWD (Chen et al., 2014), the specific antidepressant mechanism has not been elucidated.

The hippocampus (HIP) is the emotional center. Hippocampal neuronal damage is closely related to the occurrence and development of depression, and protecting hippocampal neurons and reducing neuronal apoptosis is one of the important strategies for the treatment of depression (Ho et al., 2022; Zeng et al., 2023). cAMP-response element binding protein (CREB) is a protein that regulates gene transcription and inhibits neuronal apoptosis (Mo et al., 2020). Previous studies have found that CYWD can increase the expression level of CREB in the hippocampus of depressed mice (Zhihua, 2011).

Given the crucial role of neuronal damage in the pathogenesis of depression and the potential of CYWD in anti neuronal apoptosis, in this study, we first clarified the inhibitory effect of CYWD on hippocampal neuronal apoptosis through *in vivo* and *in vitro* experiments. Then, the potential active ingredients of CYWD were identified through HPLC-MS analysis, network pharmacology, and molecular docking, and key targets and signaling pathways were analyzed to predict the possible mechanism by which CYWD inhibits hippocampal neuronal apoptosis in depressed mice. Finally, the pathway was validated through *in vivo* and *in vitro* experiments. These studies will provide scientific basis for rational clinical application and new drug development of treating depression.

## 2 Materials and methods

### 2.1 Experimental reagents and instruments

CYWD was composed of eight herbal medicines: *B. chinense* DC. (Chaihu), *C. wenyujin* Y.H.Chen et C.Ling (Yujin), *Citrus reticulata* Blanco (Chenpi), *Pinellia ternate* (Banxia), *Citrus aurantium* L. (Zhishi), *Bambusa tuldoides* Munro (Zhuru), *Poria cocos* (Fuling), and *Glycyrrhiza uralensis* Fisch. (Gancao) with a weight ratio of 15:15:10:15:10:10:15:10 according to the clinical application, which were purchased from Xiang’an Hospital Affiliated to Xiamen University. The herbs were processed into freeze-dried powder. During the experiment, the freeze-dried powder was prepared into low, medium, and high dose solutions with physiological saline at concentrations of 0.5 g/mL, 1 g/mL, and 1.5 g/mL, respectively. Fluoxetine hydrochloride and Corticosterone (CORT) were brought from Sigma (United States) and CE (United States) respectively. Isoflurane (R510-22-10) was brought from Shenzhen RWD Life Science Co., Ltd. Nissl staining solution (CO117) was brought from Beyotime Biotechnology (Shanghai, China). Caspase-3-rabbit-antibody (A0214), Bax antibody (A20227), Bcl-2 antibody (A21592), AKT1 antibody (A17909), Phospho-AKT1-S473 antibody (AP0098), CREB1 antibody (A11989), Phospho-CREB1-S133 antibody (AP0333) and ABflo^®^488 Annexin V/PI Apoptosis Detection Kit (RK05875) were purchased from ABclonal (Wuhan, China). GAPDH rabbit antibody (GB11002-100). HRP-conjugated Goat anti-rabbit IgG (GB23303) were purchased from Servicebio (Wuhan, China). Cell Counting Kit-8 (HY-K0301) was purchased from MedChemExpress (New Jersey, United States), ELISA kits for dopamine (DA, MM-0626M1), adrenocorticotropic hormone (ACTH, MM-0554M1), norepinephrine (NA, MM-0876M1), and serotonin (5-HT, MM-0443M1) were purchased from Jiangsu Meimian Industrial Co.Ltd. (Yancheng, China).

### 2.2 Animal experiments

#### 2.2.1 Laboratory animals and modeling

60 female SPF-grade Balb/c mice (6–8 weeks old, 20 ± 2 g) were purchased from the Experimental Animal Center of Xiamen University (License No: XMULAC20200055) and housed in the Animal Center of Xiamen University School of Medicine under controlled conditions (20 °C–22 °C, 40%–60% relative humidity, 12 h light/dark cycle). All animals had free access to food and water and were acclimatized for 1 week before experiments. All procedures involving animals are conducted under ethical standards and the Xiamen University Laboratory Animal Ethics (Approval No: XMULAC20200055).

60 mice were randomly divided into 6 groups (n = 10 per group): control group, model group, low-dose CYWD group, medium-dose CYWD group, high-dose CYWD group, and fluoxetine group. Except for the control group, all other groups were subcutaneously injected with corticosterone (CORT, 20 mg/kg/d) for 21 days to establish the depression model. The CYWD groups were administered with CYWD at 6.5 g/kg, 13.0 g/kg, 19.5 g/kg the respective doses (0.5 times, 1.0 times and 1.5 times of standard dose) by gavage daily, while the fluoxetine group received 10 mg/kg/d fluoxetine. Body weight was measured every 7 days. Behavioral tests were conducted on the 22-23rd day. At the end of the experiments, use small animal anesthetics (R500, RWD, China) for induction anesthesia, adjust the oxygen flow rate to 1,000–2,000 mL/min, and the concentration of isoflurane to 3%–4%. Then place the mice in the induction box, and after 2–3 min of anesthesia. After checking that the righting reflex disappeared, indicating successful induction, blood was collected *via* the eyeball, and serum was stored at −80 °C. Finally, after euthanize mice with cervical dislocation, the hippocampus was dissected from six mice in each group and stored at −80 °C. The remaining mice were perfused, and the whole brain was fixed in 4% paraformaldehyde.

#### 2.2.2 Behavioral experiments

##### 2.2.2.1 Sucrose preference test

On the 20th day of model establishment, mice were provided with both 1% sucrose solution and plain drinking water. On the 21st day, the positions of the drinking bottles were swapped. Mice were water-deprived for 24 h on the 22nd day, and the test began on the 23rd day. The consumption of plain water and sucrose solution was recorded to calculate the sucrose preference ratio (sucrose solution intake/total intake × 100%).

##### 2.2.2.2 Open field test

An open box (40 cm × 40 cm × 40 cm) was used, with 50% of the floor area designated as the central zone. A digital camera was placed 2 m above to capture the movement trajectories of the mice. Mice were placed in the central area and observed for 5 min under noise levels below 65 dB. The total distance traveled, number of entries into the central area, and distance traveled within the central area were recorded.

##### 2.2.2.3 Forced swimming test

A circular bucket (diameter 15 cm, height 40 cm) was filled with water above 10 cm depth, ensuring that mice could not touch the bottom when their heads were floating on the surface. The water temperature was maintained at approximately 25 °C. Mice were placed in the bucket, and timing started for 6 min. A digital camera placed 1 m in front of the bucket recorded the immobility time of the mice in the last 5 min. Noise levels were kept below 65 dB.

#### 2.2.3 ELISA detection of neurotransmitters

ELISA was used to determine the contents of NE and ACTH corresponding neurotransmitters in hippocampal 5-HT, DA and serum. An appropriate amount of cryopreserved hippocampal tissue was put into a 2 mL centrifuge tube, PBS (pH = 7.4) homogenate was added, and the supernatant was centrifuged at 4 °C and 3500 r/min for 20 min. Place all reagents in the kit at room temperature 30 min in advance, and operate strictly according to the instructions of the ELISA kit.

#### 2.2.4 H&E staining and Nissl staining

The H&E staining procedure was as follows: the mouse hippocampal tissue was fixed in 4% histiocyte fixative solution for 24 h, routinely dehydrated, waxed, and embedded to prepare 4 μm thickness sections. The paraffin sections were dewaxed, stained with hematoxylin and eosin, and the sections were sequentially placed in absolute ethanol, n-butanol, and xylene, and sealed with neutral gum. Finally, Panoramic Digital Slide Microscanner System (VERSA200, Leica, Germany) were used to collect and analyze microscope image.

The Nissl staining procedure is as follows: the mouse hippocampal tissue paraffin sections was placed in xylene to deparaffinize three times. After soaking in absolute ethanol, 90% ethanol and 70% ethanol, washed with distilled water for 2 min. Nissl staining solution stained and washed with distilled water after 8 min. Dehydrate with 95% ethanol for 2 min, repeated the dehydration once and then clear with xylene for 5 min, repeated again and then mount with neutral gum, collected images with Panorama Digital Slide Microscanner System (VERSA200, Leica, Germany).

### 2.3 HPLC-MS analysis

One gram of freeze-dried powder of CYWD was dissolved in 5 mL of methanol, shaken, and centrifuged at 3000 r/min for 5 min. The supernatant was filtered through a 0.45 μm microporous membrane for quantitative component detection by HPLC. The chromatographic conditions were as follows: Thermal scientific^®^ C18 reverse-phase column (4.6 mm × 260 mm), gradient elution with acetonitrile-0.1% formic acid (A-B) as the mobile phase; detection wavelengths: 210, 254, 280, and 320 nm; flow rate: 1 mL/min; column temperature: 25 °C; injection volume: 10 μL. Mass spectrometry conditions included HESI, APCI, and nano-ESI ion sources, with capillary voltage at 3.80 kV and ion source temperature at 320 °C for both positive (ESI+/MS) and negative (ESI-MS) ion modes. The high-resolution liquid chromatography-mass spectrometry instrument used was the Q Exactive Nikon Eclipse C1 (Thermo Fisher Scientific, United States), and data acquisition and analysis were performed using Thermal Xcalibur Qual Browser (version 4.1.50). Compounds in CYWD were deduced based on the mass-to-charge ratios of molecular ion peaks and fragment ion peaks in the mass spectrometry.

### 2.4 Network pharmacology

Using keywords of “Chaihu,” “Yujin,” “Banxia,” “Chenpi,” “Fuling,” “Gancao,” “Zhishi,” and “Zhuru” and based on Traditional Chinese Medicine Systems Pharmacology (TCMSP, http://lsp.nwu.edu.cn/tcmspsearch.php), retrieve the composition of all drugs in the CYWD from the database, supplemented it with PubMed, Web of Science, and CNKI databases. Under the conditions of OB value ≥30% and DL value ≥0.18, further screening of drug components is conducted to ensure good oral utilization and drug likeness.

Depression-related targets were searched in Genecard (https://www.genecards.org/), OMIM (https://www.omim.org/), and TTD databases. Drug-related targets were predicted using the TCMSP database, Pubchem, and UniProt KB (http://www.uniprot.org/). A Venn diagram was constructed to identify the intersection of disease and drug targets, and the disease-drug-component-target network was visualized using Cytoscape 3.9.1. Protein-protein interaction (PPI) analysis of the intersection targets was performed using the STRING database, and core proteins were identified based on Degree, BetweennessCentrality, and ClosenessCentrality ^.^ GO function and KEGG pathway enrichment analysis (P value ≤0.05, number of target points ≥3) was conducted using Metascape (https://metascape.org/gp/index.html).

### 2.5 Molecular docking

The active ingredients with high correlation degrees and the intersection targets were selected. After adding hydrogen to the structures of the active ingredients, assigning Tripds fields and Gasteiger - Huckel charges, they were saved in MOL2 format. The three - dimensional structures of the target proteins were obtained from the PDB database, processed, and then imported into Autodock for molecular docking. The results were output as binding energy (kcal/mol) and visualized by Pymol.

### 2.6 Validation experiments *in vivo*

#### 2.6.1 Western Blot analysis of AKT, CREB, and apoptosis-related proteins in mouse hippocampus

Frozen hippocampal tissues were homogenized in lysis buffer containing PMSF using a tissue grinder (60 Hz, 120 s). The homogenate was centrifuged at 12,000 rpm for 10 min at 4 °C,and the supernatant was collected. Proteins were denatured by boiling for 10 min. Samples were separated by SDS-PAGE and transferred to PVDF membranes. The membranes were blocked and incubated with primary antibodies (diluted 1:2000 for target proteins and 1:10000 for GAPDH) at 4 °C overnight. After washing, the membranes were incubated with HRP-conjugated secondary antibodies (1:10000) for 30 min at room temperature. A chemiluminescence detection system (Tanon-5200S, Shanghai Tanon Life Science Co., Ltd., China) was use to visualize and analyze the protein bands.

#### 2.6.2 Immunofluorescence Co-Localization of apoptosis-related proteins in the hippocampus of depressed mice

Paraffin-embedded brain sections were deparaffinized, rehydrated, and subjected to antigen retrieval. After blocking with serum, the sections were incubated with primary antibodies overnight at 4 °C, followed by secondary antibodies for 1 h at room temperature. TSA amplification, DAPI staining, and microwave treatment were performed to reduce autofluorescence. The sections were mounted and imaged using a confocal microscope (Evident FV3000, Evident China, China).

### 2.7 Validation experiments *in vitro*

#### 2.7.1 Cell culture and treatment

HT22 mouse neuronal cells were divided into five experimental groups: normal group, model group, and low-, medium-, and high-dose treatment groups. Cells in the normal group were maintained in high-glucose DMEM medium, while other groups were exposed to corticosterone (CORT) at corresponding concentrations to establish neuronal apoptosis models. The treatment groups simultaneously received culture medium containing graded concentrations of the herbal drug. All cells were cultured under sterile conditions at 37 °C with 95% humidity and 5% CO_2_.

#### 2.7.2 Optimal concentration determination using CCK-8 assay

Cells were seeded at 6,000 cells/well in 96-well plates and cultured in serum-supplemented high-glucose DMEM medium for 12 h. After cell adhesion, the medium was replaced with serum-free medium for an additional 12 h incubation. Various drug concentrations were applied to different groups with six replicates per concentrate. Following 24 h intervention, 10 μL CCK-8 working solution was added to each well. After 2 h incubation in the dark, absorbance was measured at 450 nm using a multimode microplate reader (E0288, Tecan, Switzerland).

#### 2.7.3 Flow cytometric analysis of apoptosis

HT22 cells were seeded at 1 × 10^6^ cells/well in 6-well plates and cultured under standard conditions. After 24 h treatment, cells were digested with EDTA-free trypsin, centrifuged at 1,200 rpm for 5 min, and washed twice with ice-cold PBS. Cell pellets were resuspended in 1× Binding Buffer to achieve a concentration of 1 × 10^6^ cells/mL. Aliquots of 100 μL cell suspension (1 × 10^5^ cells) were transferred to flow cytometry tubes and stained with 5 μL ABflo^®^488 Annexin V and 5–10 μL propidium iodide (PI), followed by 15 min dark incubation at room temperature. Samples were diluted with 400 μL 1×Binding Buffer and analyzed using a CytoFlex S flow cytometer (Beckman Coulter, United States).

#### 2.7.4 Nissl staining of HT22 cells

Following group-specific treatments, HT22 cells grown on coverslips were fixed and subjected to Nissl staining. After staining completion, coverslips were air-dried on glass slides, mounted with neutral balsam, and dried at 37 °C for 24. Stained specimens were imaged using Panoramic Digital Slide Microscanner System (VERSA200, Leica, Germany).

#### 2.7.5 Western Blot analysis of apoptosis-related proteins

After 24 h treatment, cells were lysed with 200 μL RIPA buffer containing 2 μL PMSF on ice for 30 min. Protein extraction and analysis were performed following standard Western blot procedures as previously described.

### 2.8 Statistical analysis

The grouping data involved in this experiment were analyzed by Graphpad prism software, and the continuous data were expressed as mean ± standard deviation (x ± s) after summarizing the data, and the one-way analysis of variance (ANOVA) was used to compare between multiple groups, and P < 0.05 was considered to be statistically significant.

## 3 Results

### 3.1 HPLC-MS results for detecting the components of CYWD

**FIGURE 1:**
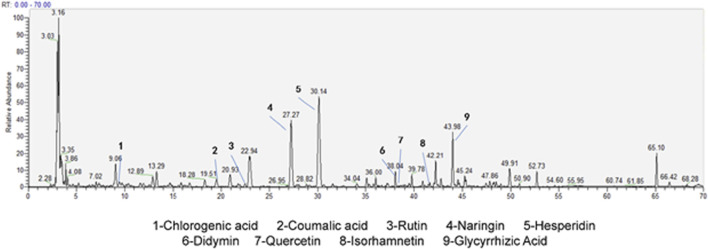
HPLC/MS of CYWD.

Through HPLC-MS technology, the detection of the components in CYWD showed that 9 components, namely, hesperidin, naringin, chlorogenic acid, coumaric acid, didymin, rutin, quercetin, isorhamnetin, and glycyrrhetinic acid, had relatively high relative abundances (Figure 1). The retention time (RT) of each component is shown in Table 1. The study not only suggests that the therapeutic effect of CYWD is the result of the combined action of multiple compounds, but also provides a basis for future network pharmacology research.

**TABLE 1:** The active component in CYWD.

Name	FW	MW	R.T. (minutes)
chlorogenic acid	C16H18O9	354	9.71
coumaric acid	C9H8O3	164	19.51
rutinoside	C27H30O16	610	22.45
naringin	C27H32O14	580	27.27
orange peel glycoside	C28H34O15	610	30.14
vanilloid glycoside	C28H34O14	594	38.04
quercetin	C15H10O7	302	38.23
isorhamnetin	C16H12O7	316	41.69
glycyrrhizic acid	C42H62O16	823	43.98

### 3.2 Pharmacodynamic results of CYWD on CORT-induced depressive model

#### 3.2.1 CYWD alleviated the depressive behaviors in mice

**FIGURE 2:**
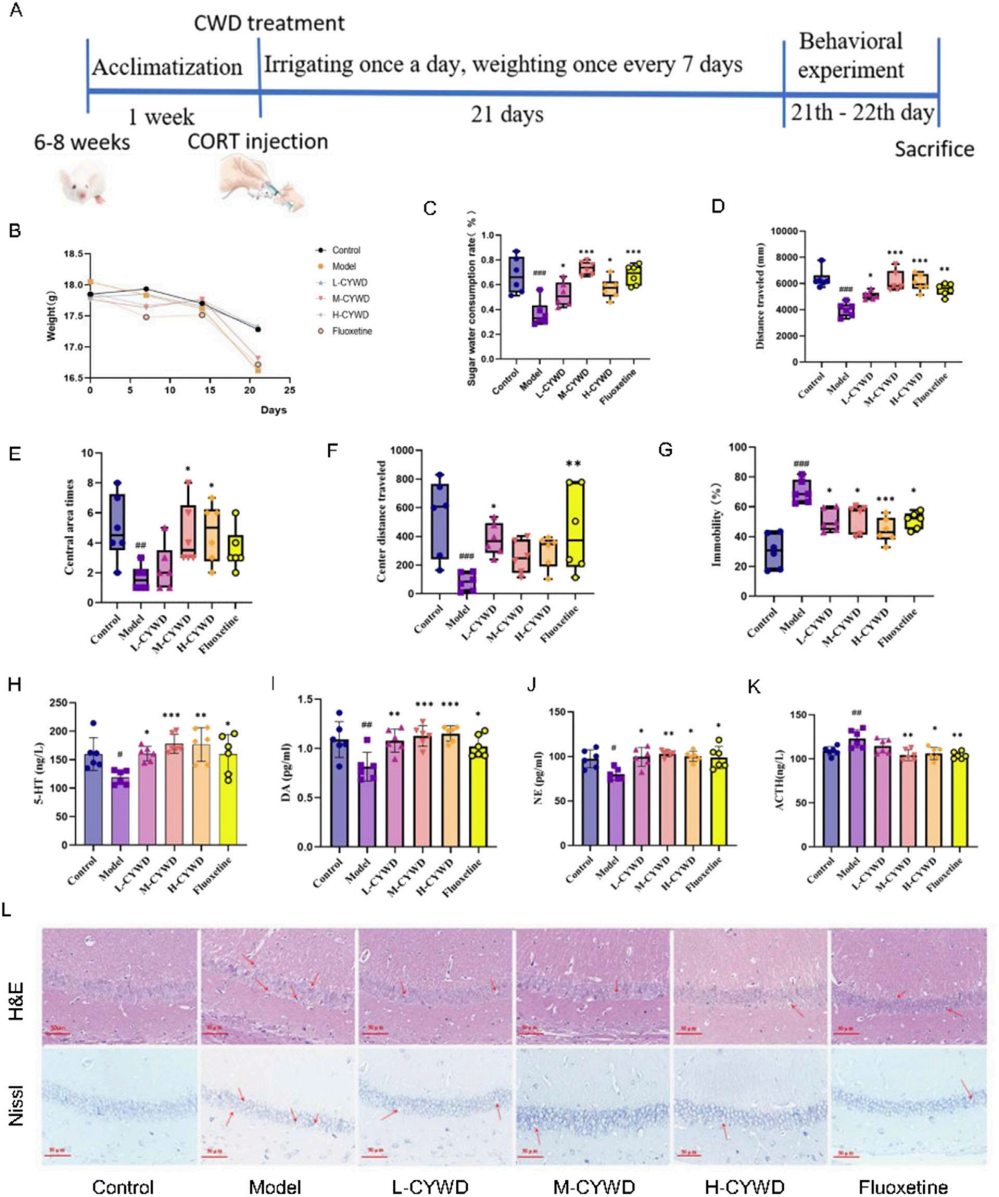
Pharmacodynamic results of CYWD on CORT-induced impression. **(A)** Schematic diagram of depressive mode, drug treatment, and pharmacodynamics indicators program schedule. **(B)** Line graph of body weight change of mice in each group during modeling. **(C)** Ratio of sugar water consumption in each group of mice in the sugar water preference Experiment. **(D)** Total distance traveled by each group of mice in the open field experiment. **(E)** The number of times each group of mice entered the central region in the open-field experiment. **(F)** Total distance traveled in the central region by each group of mice in the open-field experiment. **(G)** Percentage of immobilization time of mice in each group in the forced swimming experiment. **(H,I)** Detection of hippocampus 5-HT and DA content in each group of mice. **(J,K)** Serum NE and ACTH content of mice in each. **(L)** H&E staining and Nissl staining of hippocampus of mice in each group (X400). ^#^ *P* < 0.05, ^##^ *P* < 0.01, ^###^ *P* < 0.001 compared with the model group; *P < 0.05, **P < 0.01 compared with the normal group.

At the end of modeling, the normal mice were active and responsive, with normal diet and water intake, smooth and shiny fur. The depressive mice had reduced diet and water intake, poorer mental state, and disheveled fur. After treatment of CYWD and the positive drug (fluoxetine), all mice showed significant improvements in activity, diet and water intake, and mental state. The weight of the model group mice slightly decreased, but there was no statistical difference compared to the normal group (Figure 2B).

The effects of CYWD on depression mice were further demonstrated by behavioral tests (Figure 2A). Compared with the normal group, the model group exhibited the decreased sugar water consumption rate, the total distance of movement, the total number of entries into the central area, and the distance of movement in the central area and the increased forced swimming immobility time (Figures 2C–G). The above results indicate that the mouse depression model has been successfully established. Compared with the model group, the proportion of sugar water consumption in the medium-dose group was significantly increased (*P < 0.001*) (Figures 2C). The total exercise distance of mice in the medium-dose and high-dose groups and fluoxetine groups was significantly increased (*P < 0.05 or P < 0.01*) (Figure 2D), and the central area exercise distance of mice in the low-dose group and fluoxetine group was significantly increased (*P < 0.05 or P < 0.01*) (Figure 2E). The number of central zone entries in the high-dose group was significantly increased (*P < 0.05*) (Figure 2F). The forced swimming immobility time of mice in each administration group was significantly reduced (*P < 0.01 or P < 0.*001) (Figure 2G).

The above results suggest that CYWD treatment can improve the depression-related symptoms,and the medium-dose group has the best effect on the three behavioral changes.

#### 3.2.2 CYWD regulated neurotransmitters in depressive mice

Compared with the normal group, the model group mice showed a significant decrease in 5-HT and DA in the hippocampus (P < 0.01), a significant decrease in serum NE (P < 0.05), and a significant increase in serum ACTH (P < 0.01) (Figures 2H–K). Treatment with CYWD at medium-dose and high-dose, as well as fluoxetine, significantly increased hippocampal 5-HT, DA, and serum NE (P < 0.01 or P < 0.001), while significantly reducing serum ACTH (P < 0.05 or P < 0.01) (Figures 2H–K). Treatment with Low-dose CYWD significantly increased the levels of hippocampal 5-HT, DA, and serum NE (P < 0.05 or P < 0.01), but there was no significant change in serum ACTH (P > 0.05) (Figures 2H–K).

#### 3.2.3 CYWD alleviated hippocampal neuronal damage in depressive mice

The results of H&E staining showed that the neurons in the CA1 region of the hippocampus in the normal group were neatly arranged, the morphology was regular, and the nucleolar staining was clear. Compared with the normal group, the hippocampal neurons in the model group showed obvious necrosis, disordered arrangement, and neuronal cell atrophy or swelling, cell nucleus condensation, lysis or fragmentation. After the intervention of each administration group, the number of neurons in the low-, medium-, and high-dose groups and the fluoxetine group increased significantly, the cells were arranged regularly and evenly distributed, and the phenomenon of cell nuclei constriction, lysis or fragmentation was significantly reduced (Figure 2L). The results of Nissl staining showed that the hippocampal neurons of the normal group of mice were neatly arranged, uniform in size, and there were a large number of purple-blue Nissl bodies in the cytoplasm. In the model group, the neurons in the hippocampus were loose and disordered, the neurons were swollen, the purple-blue color became lighter, and the number of Nissl bodies in the fine cytoplasm was reduced. Compared with the model group, the neuronal arrangement of the hippocampus in the low-dose, medium-dose, and high-dose groups and the fluoxetine group was denser than that in the model group, with a darker violet-blue color and an increase in Nissl bodies, among which the most obvious changes were in the medium-dose group (Figure 2L).

### 3.3 Network pharmacology and molecular docking predicted AKT/CREB might be the key pathway for the antidepressant of CYWD

In order to explore the potential mechanism of CYWD in antidepressant treatment, we conducted network pharmacology and molecular docking studies. After screening, 134 effective ingredients were identified, including Chaihu (Gammoh et al., 2023), Yujin (Wang et al., 2021), Banxia (Chen et al., 2025), Chenpi (Ho et al., 2022), Gancao (92), Fuling (Wang et al., 2021), and Zhishi (Shukla et al., 2024). Some ingredient information is shown in Table 2.

**TABLE 2:** Part of components of CYWD.

Mol ID	Molecule Name	OB (%)	DL	Source
MOL004609	Areapillin	48.96	0.41	Chaihu
MOL013187	Cubebin	57.13	0.64	Chaihu
MOL004306	Zedoalactone B	103.59	0.22	Yujin
MOL004311	Zedoarolide A	87.97	0.3	Yujin
MOL004313	Zedoarolide B	135.56	0.21	Yujin
MOL000283	Ergosterol peroxide	40.36	0.81	Fuling
MOL000300	dehydroeburicoic acid	44.17	0.83	Fuling
MOL002311	Glycyrol	90.78	0.67	Gancao
MOL004891	shinpterocarpin	80.3	0.73	Gancao
MOL002776	Baicalin	40.12	0.75	Banxia
MOL000449	Stigmasterol	43.83	0.76	Banxia
MOL005815	Citromitin	86.9	0.51	Chenpi
MOL005828	nobiletin	61.67	0.52	Chenpi
MOL013435	poncimarin	63.62	0.35	Zhishi
MOL005828	nobiletin	61.67	0.52	Zhishi

**FIGURE 3:**
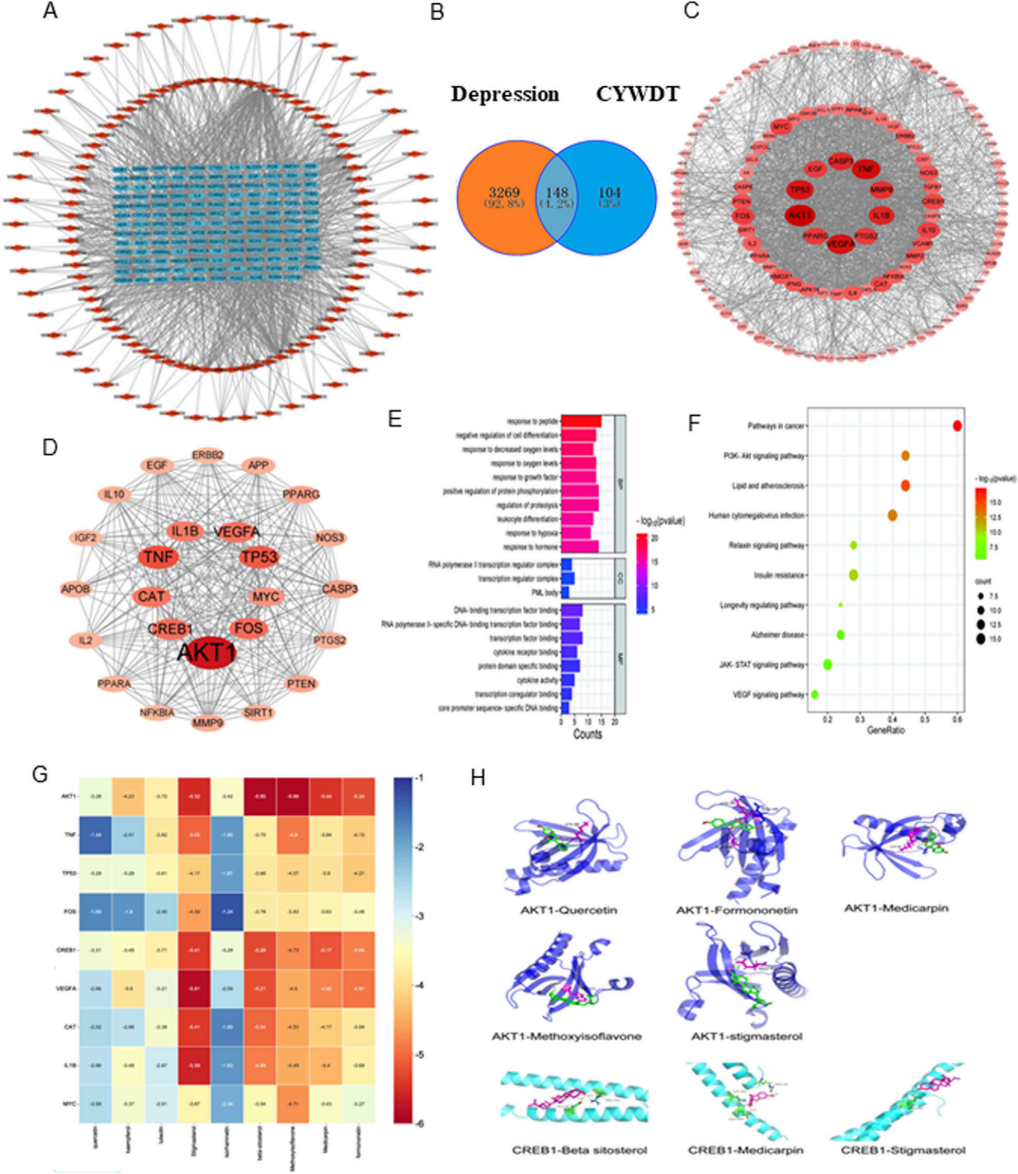
Network pharmacology and molecular docking predicted that the AKT CREB pathway might be the key pathway for the antidepressant effect of CYWD. **(A)** The relationship between the intersecting targets and the components, the blue part is the intersecting targets and the red part is the drug components. **(B)** The intersecting target points of each drug and depression in Chatyu Wendan Decoction **(C)** Protein-protein interactions between the insecting targets of Chayu Wendan Decoction and depression, with the darker red color representing the sanger carrelation. **(D)** The care protein after screening, the darker red color means the stronger its comition **(E)** Bubble plots for KEGG analysis **(F)** The grouped graph of GO enrichment analysis **(G)** Decking heat. **(H)** AKTI and CREBI decking pattern image.

Subsequently, the targets of these active components were obtained from the TCMSP database, and 252 protein targets were obtained after removing the non-target components and deduplication. At the same time, 3417 decompressive disease targets were collected from Genecard, OMIM, and TTD databases. A Venn diagram was plotted using venny2.0 to find 148 intersecting targets (Figure 3B), results are shown in Table 2, and a component-target network of 268 nodes and 1483 edges (Figure 3A) was constructed, with the outer red rhomboid as the main active component and the blue rectangle in the middle as the intersecting protein target.

Next, the intersecting targets were uploaded to the STRING database to construct a preliminary protein-protein interaction (PPI) network, which was optimized using Cytoscape (Figure 3C). 25 core proteins were screened out through three degree values, namely, Degree, Betweenness Centrality, and Closeness Centrality (Figure 3D).

The online tool Metascape was used to conduct Gene Ontology (GO) functional and Kyoto Encyclopedia of Genes and Genomes (KEGG) pathway enrichment analyses on these 25 core intersection targets (P ≤ 0.05). and 508 items (488 in Biological Process, 4 in Cellular Component, and 16 in Molecular Function) were obtained by GO enrichment, mainly focusing on peptide reaction and cell differentiation regulation (Figure 3E). 12 signaling pathways (with the number of target enrichment in each pathway being greater than 3) were obtained by the KEGG pathway enrichment analysis, and a bubble plot was drawn (Figure 3F), suggesting that its antidepressant effect may be related to pathways such as PI3K-AKT and JAK-STAT.

Finally, based on the previous analysis, molecular docking was performed on 9 active ingredients, namely, quercetin, kaempferol, luteolin, stigmasterol, isorhamnetin, β-sitosterol, isoflavone, pterocarpin, and formononetin, and the top 9 target proteins in the PPI network, namely, AKT1, TNF, TP53, CREB1, FOS, VEGFA, CAT, IL1B, and MYC (Table 3). The docking heatmap (Figure 3G) shows that each active ingredient is well docked with targets such as AKT1 and CREB1. The lower the binding energy, the higher the degree of binding, and the redder the color. The best docking result for each ingredient is shown in the docking mode diagram (Figure 3H).

**TABLE 3:** Binding energy from molecular docking analysis (kcal/mol).

Target Component	AKT1	TNF	TP53	FOS	CREB1	VEGFA	CAT	IL1B	MYC
Quercetin	−3.28	−1.44	−3.29	−1.69	−3.31	−2.66	−2.52	−2.66	−2.58
Kaempferol	−4.23	−2.41	−3.28	−1.8	−3.48	−3.9	−2.68	−3.84	−3.37
Luteolin	−3.72	−3.82	−3.61	−2.45	−3.71	−3.21	−3.39	−2.87	−2.91
Stigmasterol	−5.52	−5.05	−4.17	−4.59	−5.41	−5.81	−5.41	−5.59	−3.67
Isorhamnetin	−3.42	−1.85	−1.87	−1.24	−3.29	−2.59	−1.65	−1.82	−2.04
Beta-sitosterol	−5.85	−3.75	−3.68	−3.78	−5.26	−5.21	−5.04	−4.86	−3.54
Methoxyisoflavone	−5.88	−4.9	−4.07	−3.83	−4.73	−4.5	−4.53	−4.49	−4.71
Medicarpin	−5.44	−3.84	−3.9	−3.63	−5.17	−4.82	−4.17	−4.4	−3.43
Formononetin	−5.24	−4.15	−4.21	−3.48	−4.84	−4.91	−3.84	−3.69	−3.27

### 3.4 CYWD activated the AKT/CREB pathway in the hippocampus of depressed mice through decreasing Caspase 3 and Bax and increasing Bcl-2

**FIGURE 4:**
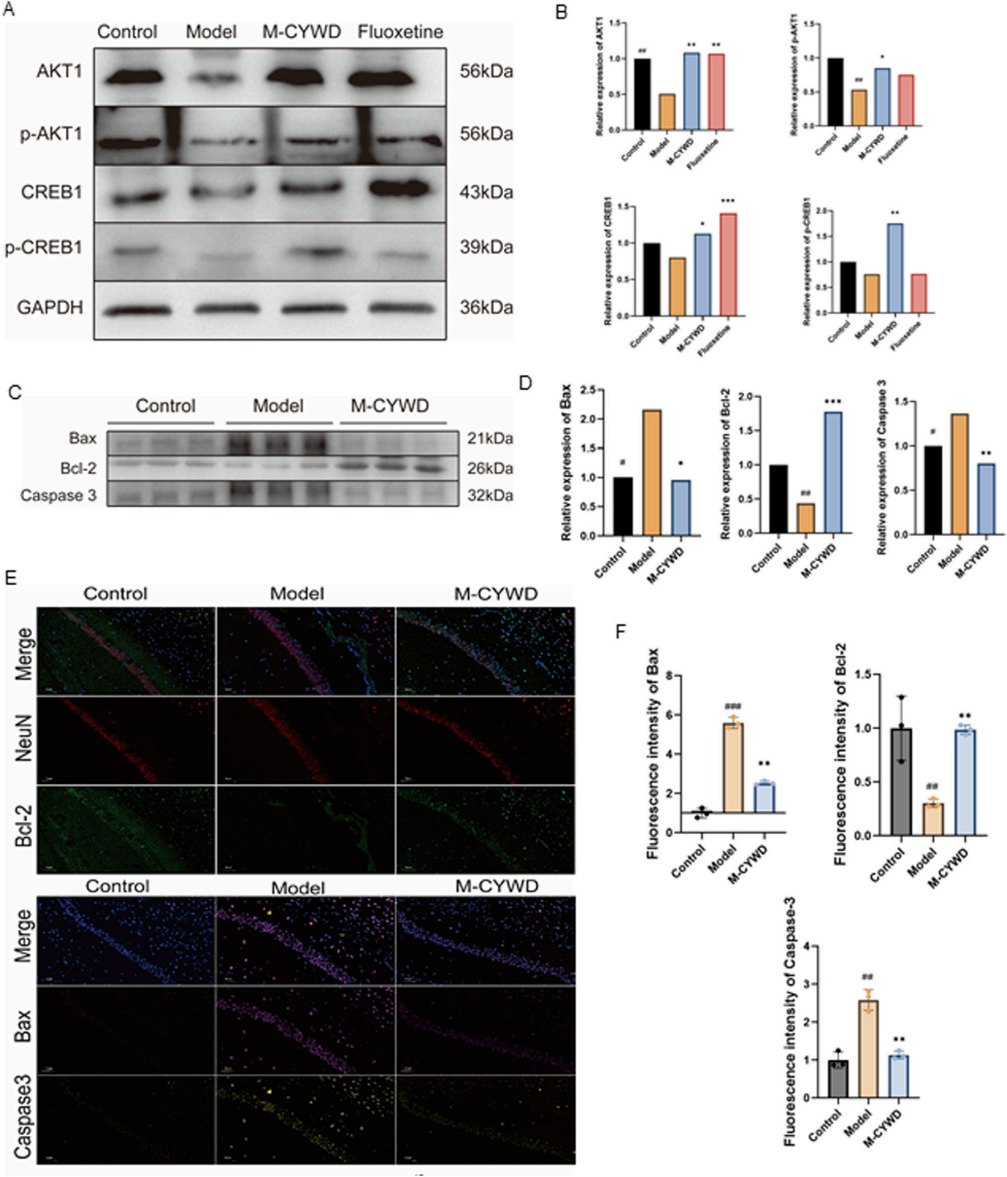
Expression of AKT-CREB pathway-related proteins and apoptosis-related proteins in the hippocampus of mice in each group. **(A)** AKT1, p-AKT1, CREB1, p-CREB1 expression in hippocampus of mice in each group. **(B)** Statistics of AKT1, p-AKT1, CREB1, p-CREB1 expression in hippocampus of mice in each group. **(C)** Expression of apoptosis-related proteins Bax, Bcl-2, and Caspase 3 in the hippocampus of mice in each group. **(D)** Statistics of apoptosis-related protein Bax, Bcl-2, and Caspase 3 expression in the hippocampus of mice in each group. **(E)** Immunofluorescence assay to detect apoptosis-related proteins: Bax, Bcl-2, Caspase 3 in the hippocampus of each group of mice and labeling hippocampal neurons with NeuN (×400). **(F)** Statistics of the average fluorescence intensity values of Bax, Bcl-2, and Caspase 3 in the hippocampus of mice in each group. ^#^ *P* < 0.05, ^##^ *P* < 0.01, ^###^ *P* < 0.001 compared with the model group; *P < 0.05, **P < 0.01 compared with the normal group.

The results of Western Blot showed that, compared with the normal group, the expression levels of AKT1 protein and p-AKT protein in the hippocampus of the model group mice were significantly decreased (P < 0.01, P < 0.05); the expression levels of CREB1 protein and p-CREB protein decreased, but there was no statistical difference. Compared with the model group, the expression levels of AKT1 protein, p-AKT protein, CREB1 protein and p-CREB1 protein in the hippocampus of mice with medium-dose CYWD were significantly increased (P < 0.01, P < 0.05, P < 0.05, P < 0.01), while in the hippocampus of mice with fluoxetine, only the expressions of AKT1 protein and CREB1 protein were significantly increased (P < 0.01, P < 0.01) (Figures 4A,B).

The results of Western Blot also showed that: compared with t the normal group, the expressions of Bax and Caspase 3 proteins in the hippocampus of the model group were significantly increased (P < 0.05), and the expression of Bcl-2 protein was significantly decreased (P < 0.01). Treatment with the medium-dose CYWD significantly decreased the expression of Bax protein and the expression of Bax protein (P < 0.05, P < 0.01), while significantly increased the expression of Bcl-2 (P < 0.001) (Figures 4C,D).

Finally, the above results were verified again by immunofluorescence detection.

Compared with the normal group, the expression of Bax protein and Caspase 3 protein in the hippocampus of the model group was significantly increased (P < 0.001, P < 0.01), and the expression of Bcl-2 protein was significantly decreased (P < 0.01) (Figures 4E,F). After the treatment of the medium-dose CYWD, the expressions of Caspase 3 and Bax proteins in the hippocampus were significantly decreased (P < 0.01 or P < 0.001), and the expression of Bcl-2 was significantly increased (P < 0.01) (Figures 4E,F).

### 3.5 CYWD inhibited the apoptosis of HT22 cells through regulating the expression of pro-apoptotic and anti-apoptotic proteins

**FIGURE 5:**
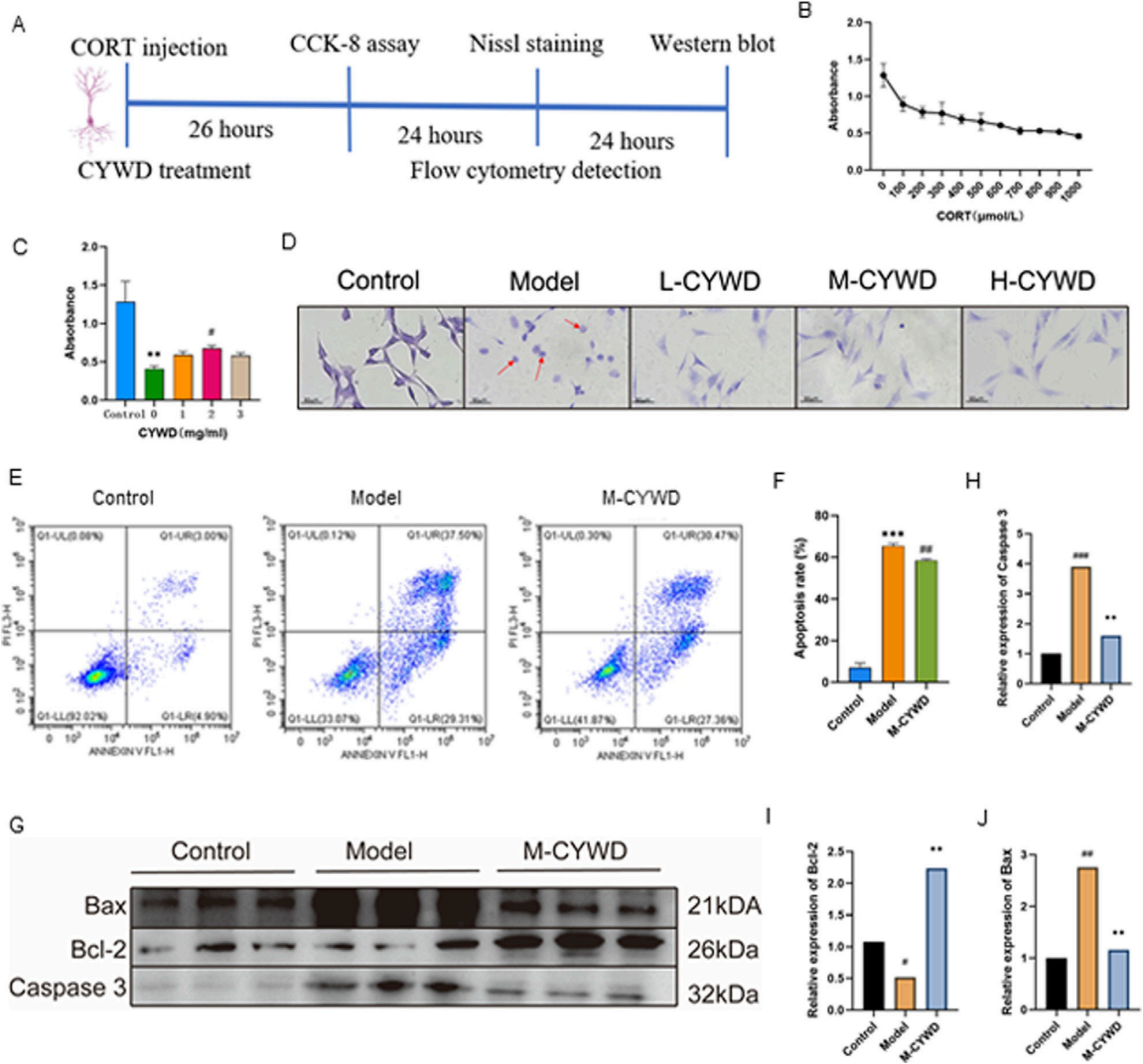
Results of the Validation Experiments in vitro. **(A)** Schematic diagram of cellular experiment. **(B)** Shows the best modeling concentration of HT22 cells with CORT intervention detected by CCK-8. **(C)** The optimal therapeutic concentration of CYWD under CORT intervention detected by cck-8. ^#^ *P* < 0.05 compared with the model group: **P < 0.01 compared with the normal group. **(D)** The Nissl staining of cells in each group under microscope (×400). **(E)** The flow cytograms of cells in normal group, model group and CYWD group. **(F)** The apoptosis rate statistics. ^##^ *P* <0.01 compared with the model group; ***P<0.001 compared with the normal group. **(G)** Graph of Bax, Bcl-2, Caspase 3 protein expression in each group of HT22 cells. **(H–J)** Statistics of Bax, Bcl-2, Caspase 3 protein expression of cells in each group. ^##^ *P* < 0.01, ^###^ *P* < 0.001 compared with the model group; *P < 0.05, **P < 0.01, ***P < 0.001 compared with the normal group.

The schematic diagram of cellular experiment is shown in Figure 5A at the beginning of paragraph. The results of CCK-8 assay showed that CORT had a significant cytotoxic effect on HT22 at a concentration of 600 μ mol/L (P < 0.01), which will be used for subsequent experimental modeling (Figure 5B). The concentrations of CYWD in the low-dose, medium-dose and high-dose groups were set as 1 mg/mL, 2 mg/mL and 3 mg/mL respectively (hereinafter referred to as the low-dose CYWD group, the medium-dose CYWD group and the high-dose CYWD group). The intervention of CYWD had no significant effect on the viability of HT22 cells, but could improve the viability of HT22 cells after CORT intervention, and the medium-dose CYWD group had the best effect. Therefore, 2 mg/mL was determined as the effective concentration for subsequent experimental research (Figure 5C).

Nissl staining showed that HT22 cells in the model group after CORT intervention had a large number of cell ruptures, a significant decrease in the number of surviving cells, retraction of neurites, a remarkable reduction in Nissl bodies within the cells, and a large number of fragmented Nissl bodies. After treatment with different doses of CYWD, the rupture of neuronal cells decreased, the neurites increased, and the number of Nissl bodies was significantly higher than that in the model group (Figure 5D). Flow cytometry detection showed that CORT could significantly increase the apoptosis rate of HT22 cells (P < 0.001) (Figures 5E,F). Intervention with the medium-dose CYWD could significantly reduce the cell apoptosis rate (Figures 5D,F).

The results of Western blot showed that compared with the normal group, the expression of Caspase 3 and Bax proteins in the model group was significantly increased (P < 0.001), and the expression of Bcl-2 protein was significantly decreased (P < 0.05); Compared with the model group, the expression of Bax protein and Caspase 3 protein in the middle-dose CYWD group were significantly decreased (P < 0.001, P < 0.001), Bcl-2 expression was significantly increased (P < 0.001) (Figures 5G–J).

The results of *in vitro* experiments showed that CYWD could promote the proliferation of HT22 cells damaged by CORT. By reducing Bax and Caspase 3, increasing Bcl-2, and inhibiting their pathological apoptosis, CYWD exerted a neuroprotective effect.

## 4 Discussion

Depression belongs to “Yu disease” in traditional Chinese medicine. The most common pathogenesis of it is liver qi stagnation and phlegm obstruction. Therefore, CYWD is composed of Wendan Decoction plus *B. chinense* DC. (Chaihu) and *C. wenyujin* Y.H.Chen et C.Ling (Yujin). Wendan Decoction comes from *Synopsis of the Three Causes and Symptoms of Diseases* with a history of over 1,600 years, which has the effects of regulating qi and resolving phlegm, and harmonizing the stomach and benefiting the gallbladder. An evaluation of a new synthetic external control method showed that the clinical efficacy of Wendan Decoction is comparable to that of 5 antidepressants, and is significantly higher than that of the other 4 antidepressants (Yang et al., 2023). Both Chaiqin Wendan Decoction (Sun et al., 2020) and Huanglian Wendan Decoction (Jia et al., 2018) have antidepressant effects. Bupleurum chinense and Curcuma aromatica are a well-known herb pair for soothing the liver and relieving depression. Animal experiments have also confirmed that both *B. chinense* DC (Bi et al., 2025; Chen et al., 2025) and *C. wenyujin* Y.H.Chen et C.Ling (Jaseela et al., 2016; Wang et al., 2021) have antidepressant effects. Therefore, adding *B. chinense* DC. and *C. wenyujin* Y.H.Chen et C.Ling to Wendan Decoction in CYWD can enhance the effects of regulating qi and resolving phlegm, as well as soothing the liver and relieving depression, which is more in line with clinical needs. In this study, a mouse model of CORT-induced depression was used to evaluate the therapeutic effect of CYWD on depression. After administering CYWD and fluoxetine, significant improvements in the depressive behavior and neurotransmitter levels of the mice were observed. These pharmacodynamic findings provided strong support for the use of CYWD in the treatment of depression.

The component analysis of CYWD by HPLC-MS technology shows that nine substances, namely, hesperidin, naringin, chlorogenic acid, coumaric acid, melittoside, rutin, quercetin, isorhamnetin, and glycyrrhizic acid, have relatively high abundances. Among these 9 components, many have been proven to have antidepressant effects. For example, quercetin relieves the depression-like behaviors of rats in a perimenopausal depression model by regulating the hypothalamic ferroptosis pathway mediated by acetylation-H3K9 (Wang et al., 2024). Isorhamnetin can effectively restore the depletion levels of Nrf2, BDNF, and HO-1 in the cerebral cortex caused by LPS-induced depression, thus enhancing the therapeutic effect of the conventional antidepressant escitalopram (Gammoh et al., 2023). The effect of CYWD in protecting against hippocampal neuronal damage may be the result of the combined action of multiple compounds in it.

In order to avoid aimless exploration, we used network pharmacology combined with molecular docking technology to predict the key targets and pathways by which CYWD inhibits hippocampal neuronal injury, and elucidate the therapeutic mechanism of the combined action of multiple components in CYWD. Network pharmacology showed that the treatment of depression with CYWD is more likely related to the PI3K/AKT signaling pathway. In the protein-protein interaction relationship, AKT1, CREB1, and Caspase 3 were the core proteins and showed relatively high degree values. At the same time, apoptosis-related proteins Bcl-2 and Bax also appeared in disease-drug intersection targets. The results of molecular docking showed that The results of molecular docking showed that the nine compounds (quercetin, kaempferol, luteolin, stigmasterol, isorhamnetin, β-sitosterol, isoflavones, pterosandne, and miscanthin) with high correlation in the drug target pattern diagram have a high degree of binding to AKT1 and CREB1. In summary, we speculated that CYWD may inhibit neuronal apoptosis by activating the AKT/CREB pathway and regulating proteins such as Bcl-2, Bax, and Caspase 3, so as to protect hippocampal neurons and play a role in the treatment of depression.

The hippocampus is located in the medial temporal lobe and is mainly responsible for memory consolidation, spatial navigation, and emotional regulation. Both animal studies and human studies have shown a close relationship between hippocampal neuron damage and depression. Imaging tests show that patients with depression often experience a reduction in hippocampal volume, which is positively correlated with the severity of the disease (Liu et al., 2020; He et al., 2021). In animal models of depression, it has also been found a decrease in the number and irregular morphology of hippocampal neurons, such as irregular cell boundaries and nuclear atrophy (Chakraborty et al., 2019; Chang et al., 2024). Nissl bodies are cytoplasmic bodies within neuronal cells, which are unique structures within neuronal cell bodies and dendrites. Nissl bodies are particularly abundant in neurons with vigorous metabolic functions. When neurons are damaged or excessively fatigued, Nissl body can decrease, disintegrate, or even disappear. Therefore, the Nissl body can serve as a marker of neuronal functional status. In the depressed mice, a significant reduction in Nissl bodies can also be observed, indicating impaired neuronal function (Shukla et al., 2024) Effective antidepressant treatment can reverse hippocampal neuronal damage. For example, exercise can reduce the apoptosis rate of hippocampal cells, promote the growth of hippocampal cells, increase the volume of the hippocampus, improve learning, memory, and emotional regulation abilities, and thus exert an antidepressant effect (Liang et al., 2019). Esketamine protects hippocampal neurons by increasing synaptic connectivity and function. SEP-363856 can significantly improve the depression-like behaviors induced by chronic unpredictable mild stress (CUMS) through reversing hippocampal neuron damage (Li et al., 2024). The *in vitro* and *in vivo* experimental results of this study showed that CORT induced depression in mice not only resulted in a decrease in the number of hippocampal neurons, but also exhibited structural abnormalities, with a significant reduction in the number of Nissl bodies in the cytoplasm. These results were consistent with existing research results. CYWD can significantly increased the number of hippocampal neurons and Nissl bodies in depressed mice, with regular and evenly distributed cell arrangement and reduced nuclear condensation, dissolution, or fragmentation. The above results indicated that CYWD had the effect of reducing hippocampal neuron damage.

Apoptosis is an important pathway for hippocampal neuronal damage (Liu et al., 2020). The AKT/CREB signaling pathway plays an important regulatory role in cell apoptosis. AKT (protein kinase B) and CREB (cAMP response element-binding protein) are important signaling molecules within cells. AKT, also known as phosphokinase B (PKB), is a key factor regulating the growth of neuronal cells and can inhibit cell apoptosis by regulating the expression of pro apoptotic and anti apoptotic genes (He et al., 2021; Fu et al., 2024).

AKT requires dual phosphorylation to be activated. When a cells receives a signal from a growth factors, PI3K is activated and phosphorylates PIP2 to generate PIP3. AKT is recruited near the cell membrane by binding to PIP3 through its PH domain. On the cell membrane, the Thr308 site of AKT is phosphorylated by PDK1 (Klein-Goldberg et al., 2025; Wei et al., 2019; Lee et al., 2025). The activated AKT triggers a cascade reactions of downstream signaling molecules through phosphorylation, thereby regulating various physiological functions of cells, such as inhibiting apoptosis and regulating metabolism (Tang et al., 2025; Li et al., 2025).

CREB is a protein located in the nucleus of eukaryotic cells. Its function is to regulate gene transcription, so it is also known as a nuclear factor that regulates transcription. CREB is one of the downstream factors of AKT, which mainly acts on the processes of learning and memory. Inducing the expression of CREB in the hippocampus helps to improve the therapeutic effect on depression (Tang et al., 2024). CREB is also involved in the formation of long-term memory and is related to the plasticity of neuronal synapses (Pittenger et al., 2002). However, CREB must be phosphorylated to form pCREB before it can function as a transcription activator. AKT can promote the phosphorylation of CREB. The phosphorylated CREB can effectively inhibit the apoptosis of neuronal cells. Elevated expression of p-CREB and CREB can reduce neuroinflammation, promote the recovery of damaged nerves, and play a neuroprotective role (Mahmoud et al., 2023; Zhang et al., 2023).

Endogenous apoptosis in cells may be influenced by multiple pathways, but ultimately focuses on the activation of Bcl-2 family proteins that control the integrity of the mitochondrial membrane. The Bcl-2 protein family consists of pro apoptotic proteins and anti apoptotic proteins. Pro apoptotic proteins include Bax and Bak, while anti apoptotic proteins include Bcl-2, Bcl xl, Mcl-1, etc (Hollville et al., 2019; Fricker et al., 2018). If t here is an imbalance in the ratio of pro-apoptotic proteins (such as Bax) to anti-apoptotic proteins (such as Bcl-2), it will affect cell fate through both anti apoptotic and pro apoptotic pathways. In healthy cells, Bax mainly exists as inactive cytoplasmic monomers or self-inhibited homodimers. Under cellular stress, Bax and Bak In healthy cells, Bax mainly exists as inactive cytoplasmic monomers or self-inhibited homodimers. Under cellular stress conditions, Bax and Bak oligomerize and insert into the mitochondrial outer membrane (MOM), leading to mitochondrial outer membrane permeabilization (MOMP) and the release of cytochrome C (Cyt C) and other proteins (Spitz and Gavathiotis, 2022).In the cytoplasm, Cyt C forms an apoptosome together with Apaf-1 and pro-Cas 9. The formation of this apoptosome activates caspase-9 (Caspase 9), which in turn activates caspase-3 (Caspase 3) (Pembe et al., 2021). Studies have shown that Caspase 3 is the most important terminal protease during the process of apoptosis. Once Caspase 3 is activated, it will initiate apoptosis (Jiang et al., 2020). After AKT activates CREB, CREB can induce the expression of anti apoptotic proteins (such as Bcl-2, Bcl xL), thereby inhibiting apoptosis. When the AKT/CREB signaling pathway is inhibited, the expression of pro apoptotic proteins (such as Bax, caspase-3) increases, while the expression of anti apoptotic proteins (such as Bcl-2) decreases, leading to increased mitochondrial outer membrane permeability, release of cytochrome C, and activation of caspase-3, thereby inducing cell apoptosis (Xu et al., 2024).

This study found that CYWD not only promotes the expression levels of AKT1 protein, p-AKT protein, CREB1 protein, and p-CREB1 protein in the hippocampal neurons with CORT-induced damage, but also activates the expression of the anti-apoptotic protein Bcl-2 while inhibiting the expressions of the pro-apoptotic proteins Bax and Caspase 3. It significantly downregulates the ratio of pro-apoptotic proteins in CORT-induced hippocampal neurons damage, verifying the prediction of network pharmacology and molecular docking that “CYWD may treat depression by inhibiting neuronal apoptosis through activating the AKT/CREB signaling pathway.”

Despite the above findings, our research still has limitations. AKT is activated by PI3K, which is itself activated by several upstream signaling pathways such as insulin receptors, receptor tyrosine kinases, G protein coupled receptors, cytokine receptors, *etc.* Once activation, ATK targets several downstream molecules and alters molecular activity through phosphorylation or formation of complexes. Based on the prediction results of network pharmacology and molecular docking, as well as the neuronal damage in depressed mice, this study only detected AKT, CREB and apoptosis-related factors, without delving deep into its upstream and downstream pathways. In the future exploration of the treatment of depression by CYWD, it may be possible to extract the active ingredients according to the results of HPLC-MS analysis, and conduct an in-depth exploration of the upstream and downstream mechanisms by which CYWD prevents the apoptosis of hippocampal neurons through the AKT/CREB signaling pathway.

## 5 Conclusion

**FIGURE 6:**
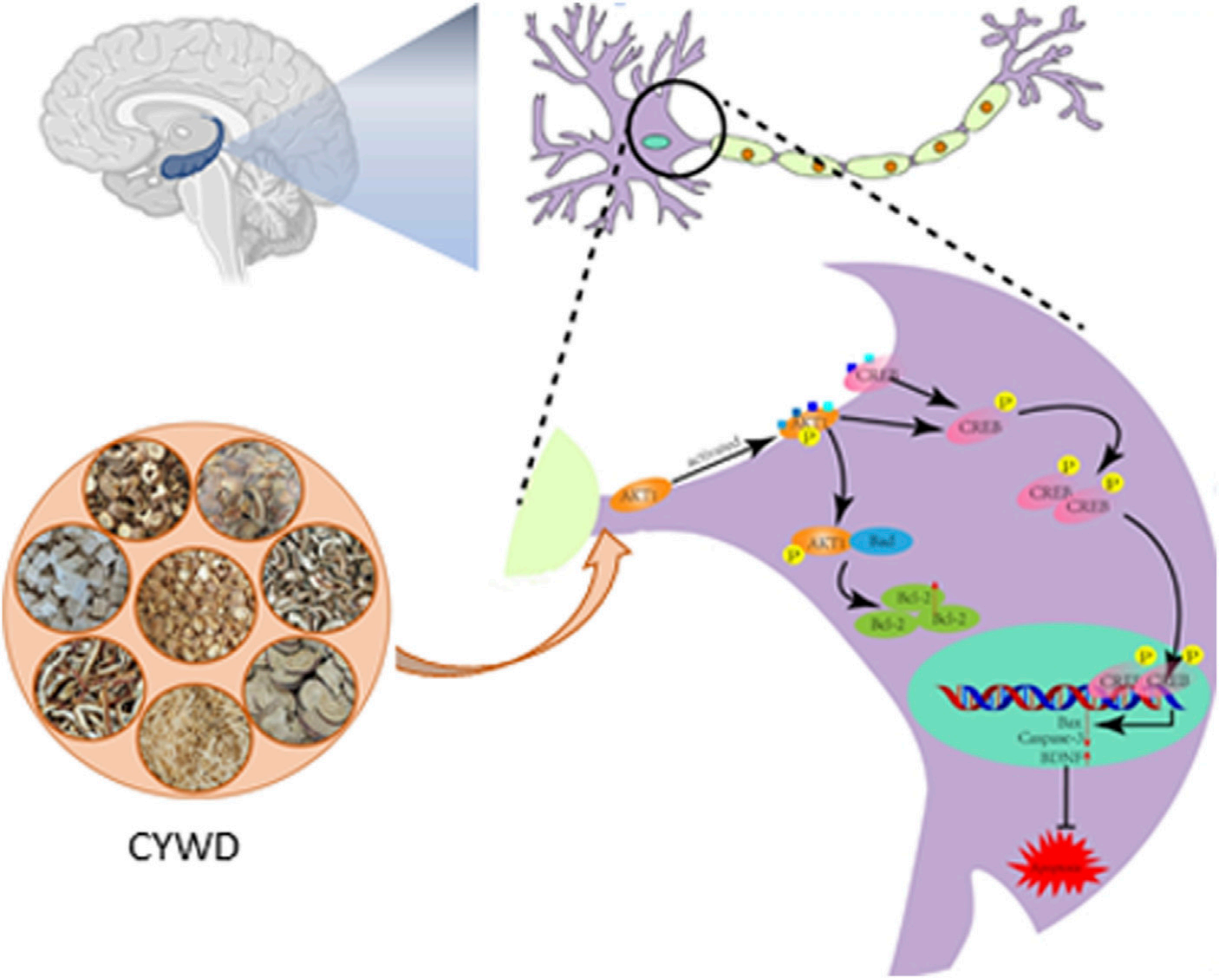
Chaiyunda Decoction’s inhibition of Hippocampal Naswan Apoptosis to Aleviating Depressionby Activating the AKT CREB Pathway.

In summary, we first confirmed that CYWD has a protective effect on the damage of hippocampal neurons in CORT-induced depressive mice. Then, combined with network pharmacology and molecular docking predictions, and validated through *in vivo* and *in vitro* experiments, CYWD can activate the AKT/CREB signaling pathway, upregulate the expression ratio of anti-apoptotic proteins, inhibit hippocampal neuron apoptosis, and reduce CORT induced hippocampal neuron damage (Figure 6). This may be the key mechanism of CYWD’s antidepressant effect and can also serve as a basis for its widespread clinical application.

## Data Availability

The original contributions presented in the study are included in the article/Supplementary Material, further inquiries can be directed to the corresponding authors.
