# Supplementary material for: Study on Chaiyuwendan decoction’s inhibition of hippocampal neuron apoptosis to alleviating depression by activating the AKT/CREB pathway
Source: Front Pharmacol. 2025 Oct 27;16:1610899. doi: 10.3389/fphar.2025.1610899 (PMC12598031; doi:10.3389/fphar.2025.1610899)
Supplement: Supplementary file 1 [file Table1.docx]

| **Supplemental Table 1 Intersecting targets between CYWD and depression** | | | | | | | |
| --- | --- | --- | --- | --- | --- | --- | --- |
| SLC6A4 | NR3C2 | ADH1C | TGFB1 | ADRA1A | PLAT | PPP3CA | CHRM3 |
| GRIA2 | IL2 | PPARG | ACHE | F3 | PPARA | DDB1 | TOP1 |
| CASP3 | AKT1 | ABCC9 | EGF | CD40LG | MAP3K7 | TIMP1 | NOX4 |
| CHRM2 | DRD1 | PTPN22 | MMP1 | ERBB2 | NFE2L2 | LTA4H | MDM2 |
| OPRM1 | CYP1A2 | ADRA2A | CDKN1C | CHRNA2 | GABRA6 | CASP8 | FGFR4 |
| MMP9 | CYP3A4 | GABRA3 | POR | CHRM1 | XDH | IGHG1 | IGFBP6 |
| GSK3B | COL1A1 | DPP4 | SERPINE1 | CHEK2 | VEGFC | ALOX5 | CCNA2 |
| SIRT1 | FOS | NOS2 | INSR | HSPA9 | CXCL10 | PTGS1 | NFATC2 |
| HTR2A | APP | GABRA5 | OPRD1 | PON2 | CASP9 | HSPB1 | CDC42 |
| TNF | GABRA1 | BCL2 | RUNX2 | SELE | MMP2 | PRKCB | CXCL13 |
| CRP | IL4 | GABRA2 | APOB | ADRB1 | RASA1 | ABAT | CD163 |
| HTR2C | AR | IL1A | IGF2 | SLC2A2 | TYR | PNP | HGF |
| SLC6A3 | PTEN | GSR | VCAM1 | CYP1B1 | SCN5A | CHUK | AHR |
| SLC6A2 | TP53 | PTGS2 | CYP19A1 | CTSD | PIK3CG | THBD | B3GAT1 |
| IL1B | MTND1 | ESR2 | APOD | DIO1 | BAX | COL3A1 | CHRM5 |
| CREB1 | ADIPOQ | RAF1 | HMGCR | SPP1 | PDE10A | CX3CR1 | MYC |
| IL10 | VEGFA | CAT | ADCY2 | MAPK14 | ERBB3 | NR1I2 | RXRA |
| IFNG | CHRNA7 | ADH1B | HMOX1 | ADRA2C | MTTP | NFKBIA | ADRA1B |
| HTR3A | PRKACA | NOS3 | MPO |  |  |  |  |

**Supplemental Table2 Components of CYWD identified by Network Pharmacology**

| Mol ID | Molecule Name | Molecule Weight | AlogP | OB (%) | DL | FASA | HL | Source |
| --- | --- | --- | --- | --- | --- | --- | --- | --- |
| MOL001645 | Linoleyl acetate | 308.56 | 6.85 | 42.1 | 0.2 | 0.21 | 7.48 | Chai Hu |
| MOL002776 | Baicalin | 446.39 | 0.64 | 40.12 | 0.75 | 0.36 | 17.36 | Chai Hu |
| MOL000449 | Stigmasterol | 412.77 | 7.64 | 43.83 | 0.76 | 0.22 | 5.57 | Chai Hu |
| MOL000354 | isorhamnetin | 316.28 | 1.76 | 49.6 | 0.31 | 0.32 | 14.34 | Chai Hu |
| MOL000422 | kaempferol | 286.25 | 1.77 | 41.88 | 0.24 | 0 | 14.74 | Chai Hu |
| MOL004598 | 3,5,6,7-tetramethoxy-2-(3,4,5-trimethoxyphenyl)chromone | 432.46 | 2.54 | 31.97 | 0.59 | 0.13 | 15.54 | Chai Hu |
| MOL004609 | Areapillin | 360.34 | 2.29 | 48.96 | 0.41 | 0.16 | 16.52 | Chai Hu |
| MOL013187 | Cubebin | 356.4 | 3.19 | 57.13 | 0.64 | 0.31 | 12.4 | Chai Hu |
| MOL004624 | Longikaurin A | 348.48 | 1.16 | 47.72 | 0.53 | 0.27 | 1.71 | Chai Hu |
| MOL004628 | Octalupine | 264.41 | -0.07 | 47.82 | 0.28 | 0.17 | 4.17 | Chai Hu |
| MOL004644 | Sainfuran | 286.3 | 3.38 | 79.91 | 0.23 | 0.22 | 8.58 | Chai Hu |
| MOL004648 | Troxerutin | 346.56 | 5.89 | 31.6 | 0.28 | 0.3 | 4.36 | Chai Hu |
| MOL004653 | (+)-Anomalin | 426.5 | 5.05 | 46.06 | 0.66 | 0.36 | 1.03 | Chai Hu |
| MOL004702 | saikosaponin c_qt | 472.78 | 3.71 | 30.5 | 0.63 | 0.2 | 6.12 | Chai Hu |
| MOL004718 | α-spinasterol | 412.77 | 7.64 | 42.98 | 0.76 | 0.22 | 6.46 | Chai Hu |
| MOL000490 | petunidin | 317.29 | 1.65 | 30.05 | 0.31 | 0 | 1.21 | Chai Hu |
| MOL000098 | quercetin | 302.25 | 1.5 | 46.43 | 0.28 | 0.38 | 14.4 | Chai Hu |
| MOL000358 | beta-sitosterol | 414.79 | 8.08 | 36.91 | 0.75 | 0.23 | 5.36 | Yu Jin |
| MOL000359 | sitosterol | 414.79 | 8.08 | 36.91 | 0.75 | 0.22 | 5.37 | Yu Jin |
| MOL004241 | curcolactone | 266.37 | 1.45 | 51.51 | 0.2 | 0.3 | 5.77 | Yu Jin |
| MOL004244 | (4aR,5R,8R,8aR)-5,8-dihydroxy-3,5,8a-trimethyl-6,7,8,9-tetrahydro-4aH-benzo[f]benzofuran-4-one | 264.35 | 1.27 | 59.52 | 0.2 | 0.25 | 13.62 | Yu Jin |
| MOL004253 | Curcumenolactone C | 264.35 | 1.35 | 39.7 | 0.19 | 0.33 | 8.19 | Yu Jin |
| MOL004260 | (E)-1,7-Diphenyl-3-hydroxy-1-hepten-5-one | 280.39 | 3.57 | 64.66 | 0.18 | 0.41 | 4.62 | Yu Jin |
| MOL004263 | (E)-5-Hydroxy-7-(4-hydroxyphenyl)-1-phenyl-1-heptene | 282.41 | 4.59 | 46.9 | 0.19 | 0.4 | 5.01 | Yu Jin |
| MOL004291 | Oxycurcumenol | 250.37 | 1.8 | 67.06 | 0.18 | 0.28 | 9.57 | Yu Jin |
| MOL004305 | Zedoalactone A | 266.37 | 1.26 | 111.43 | 0.19 | 0.32 | 3.69 | Yu Jin |
| MOL004306 | Zedoalactone B | 280.35 | 0.26 | 103.59 | 0.22 | 0.35 | 3.66 | Yu Jin |
| MOL004309 | zedoalactone E | 264.35 | 1.21 | 85.16 | 0.19 | 0.34 | 2.28 | Yu Jin |
| MOL004311 | Zedoarolide A | 296.35 | 0.05 | 87.97 | 0.3 | 0.35 | 4.01 | Yu Jin |
| MOL004313 | Zedoarolide B | 282.37 | 1.06 | 135.56 | 0.21 | 0.32 | 5.02 | Yu Jin |
| MOL004316 | 1,7-Diphenyl-3-acetoxy-6(E)-hepten | 308.45 | 5.24 | 48.47 | 0.22 | 0.4 | 4.59 | Yu Jin |
| MOL004328 | naringenin | 272.27 | 2.3 | 59.29 | 0.21 | 0.4 | 16.98 | Yu Jin |
| MOL001755 | 24-Ethylcholest-4-en-3-one | 412.77 | 8.18 | 36.08 | 0.76 | 0.25 | 5.49 | Ban Xia |
| MOL002670 | Cavidine | 353.45 | 3.72 | 35.64 | 0.81 | 0 | 5.78 | Ban Xia |
| MOL002714 | baicalein | 270.25 | 2.33 | 33.52 | 0.21 | 0.36 | 16.25 | Ban Xia |
| MOL002776 | Baicalin | 446.39 | 0.64 | 40.12 | 0.75 | 0.36 | 17.36 | Ban Xia |
| MOL000358 | beta-sitosterol | 414.79 | 8.08 | 36.91 | 0.75 | 0.23 | 5.36 | Ban Xia |
| MOL000449 | Stigmasterol | 412.77 | 7.64 | 43.83 | 0.76 | 0.22 | 5.57 | Ban Xia |
| MOL005030 | gondoic acid | 310.58 | 7.75 | 30.7 | 0.2 | 0.21 | 4.79 | Ban Xia |
| MOL000519 | coniferin | 314.41 | 3.16 | 31.11 | 0.32 | 0.27 | 28.1 | Ban Xia |
| MOL006936 | 10,13-eicosadienoic | 308.56 | 7.3 | 39.99 | 0.2 | 0.22 | 5.59 | Ban Xia |
| MOL006937 | 12,13-epoxy-9-hydroxynonadeca-7,10-dienoic acid | 324.51 | 4.58 | 42.15 | 0.24 | 0.25 | 2.72 | Ban Xia |
| MOL006957 | (3S,6S)-3-(benzyl)-6-(4-hydroxybenzyl)piperazine-2,5-quinone | 310.38 | 2.15 | 46.89 | 0.27 | 0.43 | 3.81 | Ban Xia |
| MOL003578 | Cycloartenol | 426.8 | 7.55 | 38.69 | 0.78 | 0 | 5 | Ban Xia |
| MOL006967 | beta-D-Ribofuranoside, xanthine-9 | 284.26 | -1.29 | 44.72 | 0.21 | 0.3 | 7.91 | Ban Xia |
| MOL000359 | sitosterol | 414.79 | 8.08 | 36.91 | 0.75 | 0.22 | 5.37 | Chen Pi |
| MOL004328 | naringenin | 272.27 | 2.3 | 59.29 | 0.21 | 0.4 | 16.98 | Chen Pi |
| MOL005100 | 5,7-dihydroxy-2-(3-hydroxy-4-methoxyphenyl)chroman-4-one | 302.3 | 2.28 | 47.74 | 0.27 | 0.31 | 16.51 | Chen Pi |
| MOL005815 | Citromitin | 404.45 | 3 | 86.9 | 0.51 | 0.14 | 15.62 | Chen Pi |
| MOL005828 | nobiletin | 402.43 | 3.04 | 61.67 | 0.52 | 0.13 | 16.2 | Chen Pi |
| MOL004941 | (2R)-7-hydroxy-2-(4-hydroxyphenyl)chroman-4-one | 256.27 | 2.57 | 71.12 | 0.18 | 0 | 18.09 | Gan Cao |
| MOL001792 | DFV | 256.27 | 2.57 | 32.76 | 0.18 | 0.42 | 17.89 | Gan Cao |
| MOL004835 | Glypallichalcone | 284.33 | 3.4 | 61.6 | 0.19 | 0 | 17.01 | Gan Cao |
| MOL004841 | Licochalcone B | 286.3 | 2.88 | 76.76 | 0.19 | 0 | 17.02 | Gan Cao |
| MOL004985 | icos-5-enoic acid | 310.58 | 7.75 | 30.7 | 0.2 | 0 | 5.28 | Gan Cao |
| MOL004996 | gadelaidic acid | 310.58 | 7.75 | 30.7 | 0.2 | 0 | 5.25 | Gan Cao |
| MOL003896 | 7-Methoxy-2-methyl isoflavone | 266.31 | 3.36 | 42.56 | 0.2 | 0.33 | 16.89 | Gan Cao |
| MOL000500 | Vestitol | 272.32 | 3.15 | 74.66 | 0.21 | 0 | 3 | Gan Cao |
| MOL004957 | HMO | 268.28 | 2.58 | 38.37 | 0.21 | 0 | 16.56 | Gan Cao |
| MOL004328 | naringenin | 272.27 | 2.3 | 59.29 | 0.21 | 0.4 | 16.98 | Gan Cao |
| MOL000392 | formononetin | 268.28 | 2.58 | 69.67 | 0.21 | 0 | 17.04 | Gan Cao |
| MOL000422 | kaempferol | 286.25 | 1.77 | 41.88 | 0.24 | 0 | 14.74 | Gan Cao |
| MOL000417 | Calycosin | 284.28 | 2.32 | 47.75 | 0.24 | 0 | 17.1 | Gan Cao |
| MOL004991 | 7-Acetoxy-2-methylisoflavone | 294.32 | 3.15 | 38.92 | 0.26 | 0 | 17.49 | Gan Cao |
| MOL004990 | 7,2',4'-trihydroxy－5-methoxy-3－arylcoumarin | 300.28 | 2.56 | 83.71 | 0.27 | 0 | 0.99 | Gan Cao |
| MOL004860 | licorice glycoside E | 693.71 | 1.59 | 32.89 | 0.27 | 0.31 | 25.39 | Gan Cao |
| MOL000098 | quercetin | 302.25 | 1.5 | 46.43 | 0.28 | 0.38 | 14.4 | Gan Cao |
| MOL000497 | licochalcone a | 338.43 | 4.62 | 40.79 | 0.29 | 0 | 16.2 | Gan Cao |
| MOL000239 | Jaranol | 314.31 | 2.09 | 50.83 | 0.29 | 0.29 | 15.5 | Gan Cao |
| MOL005016 | Odoratin | 314.31 | 2.3 | 49.95 | 0.3 | 0 | 16.35 | Gan Cao |
| MOL000354 | isorhamnetin | 316.28 | 1.76 | 49.6 | 0.31 | 0.32 | 14.34 | Gan Cao |
| MOL004898 | 5-Prenylbutein | 340.4 | 4.49 | 46.27 | 0.31 | 0.43 | 15.24 | Gan Cao |
| MOL004910 | Glabranin | 324.4 | 4.42 | 52.9 | 0.31 | 0 | 16.24 | Gan Cao |
| MOL004945 | Isobavachin | 324.4 | 4.42 | 36.57 | 0.32 | 0 | 17.95 | Gan Cao |
| MOL004848 | licochalcone G | 354.43 | 4.35 | 49.25 | 0.32 | 0.35 | 15.75 | Gan Cao |
| MOL004980 | Inflacoumarin A | 322.38 | 4.7 | 39.71 | 0.33 | 0 | 2.31 | Gan Cao |
| MOL004961 | Quercetin der. | 330.31 | 1.82 | 46.45 | 0.33 | 0 | 16.61 | Gan Cao |
| MOL002565 | Medicarpin | 270.3 | 2.66 | 49.22 | 0.34 | 0.31 | 8.46 | Gan Cao |
| MOL004829 | Glepidotin B | 340.4 | 3.88 | 64.46 | 0.34 | 0 | 15.98 | Gan Cao |
| MOL004828 | Glepidotin A | 338.38 | 3.9 | 44.72 | 0.35 | 0 | 16.09 | Gan Cao |
| MOL004815 | Kanzonol B | 322.38 | 3.96 | 39.62 | 0.35 | 0 | 16.16 | Gan Cao |
| MOL004907 | Glyzaglabrin | 298.26 | 2.1 | 61.07 | 0.35 | 0 | 21.2 | Gan Cao |
| MOL004882 | Licocoumarone | 340.4 | 4.98 | 33.21 | 0.36 | 0 | 9.66 | Gan Cao |
| MOL003656 | Lupiwighteone | 338.38 | 3.92 | 51.64 | 0.37 | 0.36 | 15.63 | Gan Cao |
| MOL005020 | dehydroglyasperins C | 340.4 | 4.3 | 53.82 | 0.37 | 0 | 2.75 | Gan Cao |
| MOL004915 | Eurycarpin A | 338.38 | 3.92 | 43.28 | 0.37 | 0 | 17.1 | Gan Cao |
| MOL004838 | 8-(6-hydroxy-2-benzofuranyl)-2,2-dimethyl-5-chromenol | 308.35 | 4.2 | 58.44 | 0.38 | 0.34 | 8.71 | Gan Cao |
| MOL005000 | Gancaonin G | 352.41 | 4.17 | 60.44 | 0.39 | 0 | 16.13 | Gan Cao |
| MOL004811 | Glyasperin C | 356.45 | 4.73 | 45.56 | 0.4 | 0 | 3.13 | Gan Cao |
| MOL004856 | Gancaonin A | 352.41 | 4.17 | 51.08 | 0.4 | 0 | 16.82 | Gan Cao |
| MOL004993 | 8-prenylated eriodictyol | 356.4 | 3.89 | 53.79 | 0.4 | 0 | 15.7 | Gan Cao |
| MOL004864 | 5,7-dihydroxy-3-(4-methoxyphenyl)-8-(3-methylbut-2-enyl)chromone | 352.41 | 4.17 | 30.49 | 0.41 | 0 | 14.99 | Gan Cao |
| MOL004989 | 6-prenylated eriodictyol | 356.4 | 3.89 | 39.22 | 0.41 | 0 | 16.52 | Gan Cao |
| MOL004863 | 3-(3,4-dihydroxyphenyl)-5,7-dihydroxy-8-(3-methylbut-2-enyl)chromone | 354.38 | 3.65 | 66.37 | 0.41 | 0 | 15.81 | Gan Cao |
| MOL004935 | Sigmoidin-B | 356.4 | 3.89 | 34.88 | 0.41 | 0 | 14.49 | Gan Cao |
| MOL004866 | 2-(3,4-dihydroxyphenyl)-5,7-dihydroxy-6-(3-methylbut-2-enyl)chromone | 354.38 | 3.92 | 44.15 | 0.41 | 0 | 16.77 | Gan Cao |
| MOL004883 | Licoisoflavone | 354.38 | 3.65 | 41.61 | 0.42 | 0 | 16.09 | Gan Cao |
| MOL004949 | Isolicoflavonol | 354.38 | 3.63 | 45.17 | 0.42 | 0 | 15.55 | Gan Cao |
| MOL004814 | Isotrifoliol | 298.26 | 2.99 | 31.94 | 0.42 | 0 | 7.91 | Gan Cao |
| MOL004913 | 1,3-dihydroxy-9-methoxy-6-benzofurano[3,2-c]chromenone | 298.26 | 2.99 | 48.14 | 0.43 | 0 | 8.87 | Gan Cao |
| MOL004849 | Licoarylcoumarin | 368.41 | 4.03 | 59.62 | 0.43 | 0 | 0.69 | Gan Cao |
| MOL004808 | glyasperin B | 370.43 | 4.02 | 65.22 | 0.44 | 0 | 16.1 | Gan Cao |
| MOL004911 | Glabrene | 322.38 | 3.77 | 46.27 | 0.44 | 0 | 3.63 | Gan Cao |
| MOL004833 | Phaseolinisoflavan | 324.4 | 3.95 | 32.01 | 0.45 | 0 | 2.66 | Gan Cao |
| MOL004857 | Gancaonin B | 368.41 | 3.91 | 48.79 | 0.45 | 0 | 16.49 | Gan Cao |
| MOL004908 | Glabridin | 324.4 | 3.95 | 53.25 | 0.47 | 0 | 0.03 | Gan Cao |
| MOL004855 | Licoricone | 382.44 | 4.16 | 63.58 | 0.47 | 0 | 16.37 | Gan Cao |
| MOL004879 | Glycyrin | 382.44 | 4.67 | 52.61 | 0.47 | 0 | 1.31 | Gan Cao |
| MOL005012 | Licoagroisoflavone | 336.36 | 3.48 | 57.28 | 0.49 | 0 | 19.64 | Gan Cao |
| MOL004912 | Glabrone | 336.36 | 3.12 | 52.51 | 0.5 | 0 | 16.09 | Gan Cao |
| MOL004820 | kanzonols W | 336.36 | 3.63 | 50.48 | 0.52 | 0 | 0.15 | Gan Cao |
| MOL004978 | 4'-Methoxyglabridin | 338.43 | 4.2 | 36.21 | 0.52 | 0 | -0.13 | Gan Cao |
| MOL004914 | 1,3-dihydroxy-8,9-dimethoxy-6-benzofurano[3,2-c]chromenone | 328.29 | 2.98 | 62.9 | 0.53 | 0 | 9.32 | Gan Cao |
| MOL004810 | glyasperin F | 354.38 | 2.97 | 75.84 | 0.54 | 0 | 15.64 | Gan Cao |
| MOL001484 | Inermine | 284.28 | 2.44 | 75.18 | 0.54 | 0.3 | 11.72 | Gan Cao |
| MOL004885 | licoisoflavanone | 354.38 | 2.97 | 52.47 | 0.54 | 0 | 15.67 | Gan Cao |
| MOL004884 | Licoisoflavone B | 352.36 | 2.85 | 38.93 | 0.55 | 0 | 15.73 | Gan Cao |
| MOL004905 | 3,22-Dihydroxy-11-oxo-delta(12)-oleanene-27-alpha-methoxycarbonyl-29-oic acid | 512.75 | 4.37 | 34.32 | 0.55 | 0 | 3.56 | Gan Cao |
| MOL004827 | Semilicoisoflavone B | 352.36 | 2.85 | 48.78 | 0.55 | 0 | 17.02 | Gan Cao |
| MOL004806 | euchrenone | 406.56 | 6.35 | 30.29 | 0.57 | 0 | 15.89 | Gan Cao |
| MOL004974 | 3'-Methoxyglabridin | 354.43 | 3.93 | 46.16 | 0.57 | 0 | 0.52 | Gan Cao |
| MOL004966 | 3'-Hydroxy-4'-O-Methylglabridin | 354.43 | 3.93 | 43.71 | 0.57 | 0 | -0.61 | Gan Cao |
| MOL005017 | Phaseol | 336.36 | 4.87 | 78.77 | 0.58 | 0 | 9.64 | Gan Cao |
| MOL005003 | Licoagrocarpin | 338.43 | 4.51 | 58.81 | 0.58 | 0.27 | 9.45 | Gan Cao |
| MOL005007 | Glyasperins M | 368.41 | 3.22 | 72.67 | 0.59 | 0 | 15.57 | Gan Cao |
| MOL005008 | Glycyrrhiza flavonol A | 370.38 | 2.17 | 41.28 | 0.6 | 0 | 13.71 | Gan Cao |
| MOL004824 | Gauflnqqcsxbpk-sfhvurjksa-n | 384.41 | 2.96 | 60.25 | 0.63 | 0 | 4.31 | Gan Cao |
| MOL004959 | 1-Methoxyphaseollidin | 354.43 | 4.25 | 69.98 | 0.64 | 0 | 9.53 | Gan Cao |
| MOL004904 | licopyranocoumarin | 384.41 | 3.04 | 80.36 | 0.65 | 0 | 0.08 | Gan Cao |
| MOL002311 | Glycyrol | 366.39 | 4.85 | 90.78 | 0.67 | 0.28 | 9.85 | Gan Cao |
| MOL005013 | 18α-hydroxyglycyrrhetic acid | 486.76 | 4.55 | 41.16 | 0.71 | 0 | 4.96 | Gan Cao |
| MOL004805 | Shinflavanone | 390.51 | 5.48 | 31.79 | 0.72 | 0.35 | 14.82 | Gan Cao |
| MOL004891 | shinpterocarpin | 322.38 | 3.46 | 80.3 | 0.73 | 0.32 | 6.5 | Gan Cao |
| MOL004903 | liquiritin | 418.43 | 0.66 | 65.69 | 0.74 | 0 | 17.96 | Gan Cao |
| MOL000359 | sitosterol | 414.79 | 8.08 | 36.91 | 0.75 | 0.22 | 5.37 | Gan Cao |
| MOL000211 | Mairin | 456.78 | 6.52 | 55.38 | 0.78 | 0.26 | 8.87 | Gan Cao |
| MOL005001 | Gancaonin H | 420.49 | 4.71 | 50.1 | 0.78 | 0 | 16.64 | Gan Cao |
| MOL004917 | glycyroside | 562.57 | -0.73 | 37.25 | 0.79 | 0 | 14.62 | Gan Cao |
| MOL004948 | Isoglycyrol | 366.39 | 4.36 | 44.7 | 0.84 | 0 | 6.69 | Gan Cao |
| MOL005018 | Xambioona | 388.49 | 4.68 | 54.85 | 0.87 | 0 | 14.5 | Gan Cao |
| MOL004988 | Kanzonol F | 420.54 | 5.3 | 32.47 | 0.89 | 0.28 | 9.98 | Gan Cao |
| MOL004924 | (-)-Medicocarpin | 432.46 | 0.75 | 40.99 | 0.95 | 0 | 13.2 | Gan Cao |
| MOL000273 | Xslkakrojkmhit-wiukaadnsa-n | 470.76 | 5.41 | 30.93 | 0.81 | 0 | 6.81 | Fu Ling |
| MOL000275 | trametenolic acid | 456.78 | 7.03 | 38.71 | 0.8 | 0 | 7.78 | Fu Ling |
| MOL000276 | 7,9(11)-dehydropachymic acid | 526.83 | 6.1 | 35.11 | 0.81 | 0 | 7.34 | Fu Ling |
| MOL000279 | Cerevisterol | 430.74 | 5.15 | 37.96 | 0.77 | 0 | 5.31 | Fu Ling |
| MOL000280 | Dehydrotumulosic acid | 484.79 | 5.72 | 31.07 | 0.82 | 0 | 7.42 | Fu Ling |
| MOL000282 | ergosta-7,22E-dien-3beta-ol | 398.74 | 7.18 | 43.51 | 0.72 | 0 | 5.11 | Fu Ling |
| MOL000283 | Ergosterol peroxide | 430.74 | 7.17 | 40.36 | 0.81 | 0 | 3.43 | Fu Ling |
| MOL000285 | Polyporenic acid C | 482.77 | 5.68 | 38.26 | 0.82 | 0 | 6.77 | Fu Ling |
| MOL000285 | 3beta-Hydroxy-24-methylene-8-lanostene-21-oic acid | 470.81 | 7.33 | 38.7 | 0.81 | 0 | 6.59 | Fu Ling |
| MOL000289 | pachymic acid | 528.85 | 6.54 | 33.63 | 0.81 | 0 | 9.27 | Fu Ling |
| MOL000290 | Poricoic acid A | 498.77 | 5.94 | 30.61 | 0.76 | 0 | 8.26 | Fu Ling |
| MOL000291 | Poricoic acid B | 484.74 | 5.64 | 30.52 | 0.75 | 0 | 8.67 | Fu Ling |
| MOL000292 | poricoic acid C | 482.77 | 7.11 | 38.15 | 0.75 | 0 | 7.73 | Fu Ling |
| MOL000296 | hederagenin | 414.79 | 8.08 | 36.91 | 0.75 | 0 | 5.35 | Fu Ling |
| MOL000300 | dehydroeburicoic acid | 453.75 | 6.35 | 44.17 | 0.83 | 0.04 | 7.04 | Fu Ling |
| MOL013276 | poncirin | 594.62 | -0.21 | 36.55 | 0.74 | 0.28 | 14.83 | Zhi Shi |
| MOL013277 | Isosinensetin | 372.4 | 3.06 | 51.15 | 0.44 | 0.14 | 15.84 | Zhi Shi |
| MOL013279 | 5,7,4'-Trimethylapigenin | 312.34 | 3.09 | 39.83 | 0.3 | 0.22 | 15.85 | Zhi Shi |
| MOL013428 | isosakuranetin-7-rutinoside | 594.62 | -0.21 | 41.24 | 0.72 | 0.27 | 14.75 | Zhi Shi |
| MOL013430 | Prangenin | 286.3 | 2.49 | 43.6 | 0.29 | 0.27 | -2.81 | Zhi Shi |
| MOL013433 | prangenin hydrate | 304.32 | 1.72 | 72.63 | 0.29 | 0.26 | -3.46 | Zhi Shi |
| MOL013435 | poncimarin | 330.41 | 2.74 | 63.62 | 0.35 | 0.33 | 2.26 | Zhi Shi |
| MOL013436 | isoponcimarin | 330.41 | 2.94 | 63.28 | 0.31 | 0.33 | 1.52 | Zhi Shi |
| MOL013437 | 6-Methoxy aurapten | 328.44 | 5.16 | 31.24 | 0.3 | 0.28 | 4.1 | Zhi Shi |
| MOL013440 | citrusin B | 568.63 | 0.02 | 40.8 | 0.71 | 0.24 | 2.07 | Zhi Shi |
| MOL001798 | neohesperidin_qt | 302.3 | 2.28 | 71.17 | 0.27 | 0 | 15.96 | Zhi Shi |
| MOL001803 | Sinensetin | 372.4 | 3.06 | 50.56 | 0.45 | 0.13 | 15.52 | Zhi Shi |
| MOL001941 | Ammidin | 270.3 | 3.65 | 34.55 | 0.22 | 0.28 | -1.35 | Zhi Shi |
| MOL013352 | Obacunone | 454.56 | 2.68 | 43.29 | 0.77 | 0.31 | -13.04 | Zhi Shi |
| MOL002914 | Eriodyctiol (flavanone) | 288.27 | 2.03 | 41.35 | 0.24 | 0.39 | 15.88 | Zhi Shi |
| MOL004328 | naringenin | 272.27 | 2.3 | 59.29 | 0.21 | 0.4 | 16.98 | Zhi Shi |
| MOL005100 | 5,7-dihydroxy-2-(3-hydroxy-4-methoxyphenyl)chroman-4-one | 302.3 | 2.28 | 47.74 | 0.27 | 0.31 | 16.51 | Zhi Shi |
| MOL005828 | nobiletin | 402.43 | 3.04 | 61.67 | 0.52 | 0.13 | 16.2 | Zhi Shi |
| MOL005849 | didymin | 286.3 | 2.55 | 38.55 | 0.24 | 0 | 16.86 | Zhi Shi |
| MOL000006 | luteolin | 286.25 | 2.07 | 36.16 | 0.25 | 0.39 | 15.94 | Zhi Shi |
| MOL007879 | Tetramethoxyluteolin | 342.37 | 3.07 | 43.68 | 0.37 | 0.17 | 15.45 | Zhi Shi |
| MOL009053 | (+)-Dehydrodiconiferyl alcohol | 358.42 | 2.16 | 50.76 | 0.39 | 0.27 | 7.58 | Zhi Shi |
